# The psychophysiology of music-based interventions and the experience of pain

**DOI:** 10.3389/fpsyg.2024.1361857

**Published:** 2024-05-10

**Authors:** Carolyn A. Arnold, Matthew K. Bagg, Alan R. Harvey

**Affiliations:** ^1^Department of Anaesthesiology and Perioperative Medicine, Monash University, Melbourne, VIC, Australia; ^2^Caulfield Pain Management and Research Centre, Alfred Health, Melbourne, VIC, Australia; ^3^School of Health Sciences, University of Notre Dame Australia, Fremantle, WA, Australia; ^4^Perron Institute for Neurological and Translational Science, Perth, WA, Australia; ^5^Centre for Pain IMPACT, Neuroscience Research Institute, Sydney, NSW, Australia; ^6^Curtin Health Innovation Research Institute, Faculty of Health Sciences, Curtin University, Bentley, WA, Australia; ^7^School of Human Sciences and Conservatorium of Music, The University of Western Australia, Perth, WA, Australia

**Keywords:** music, pain, therapy, endorphins, oxytocin, dopamine

## Abstract

In modern times there is increasing acceptance that music-based interventions are useful aids in the clinical treatment of a range of neurological and psychiatric conditions, including helping to reduce the perception of pain. Indeed, the belief that music, whether listening or performing, can alter human pain experiences has a long history, dating back to the ancient Greeks, and its potential healing properties have long been appreciated by indigenous cultures around the world. The subjective experience of acute or chronic pain is complex, influenced by many intersecting physiological and psychological factors, and it is therefore to be expected that the impact of music therapy on the pain experience may vary from one situation to another, and from one person to another. Where pain persists and becomes chronic, aberrant central processing is a key feature associated with the ongoing pain experience. Nonetheless, beneficial effects of exposure to music on pain relief have been reported across a wide range of acute and chronic conditions, and it has been shown to be effective in neonates, children and adults. In this comprehensive review we examine the various neurochemical, physiological and psychological factors that underpin the impact of music on the pain experience, factors that potentially operate at many levels – the periphery, spinal cord, brainstem, limbic system and multiple areas of cerebral cortex. We discuss the extent to which these factors, individually or in combination, influence how music affects both the quality and intensity of pain, noting that there remains controversy about the respective roles that diverse central and peripheral processes play in this experience. Better understanding of the mechanisms that underlie music’s impact on pain perception together with insights into central processing of pain should aid in developing more effective synergistic approaches when music therapy is combined with clinical treatments. The ubiquitous nature of music also facilitates application from the therapeutic environment into daily life, for ongoing individual and social benefit.

## Introduction

*“One good thing about music, when it hits you, you feel no pain” Bob Marley.*

There is increasing evidence that music-based interventions are inexpensive, safe, non-invasive and useful aids in the clinical treatment of a range of medical, neurological and psychiatric conditions, including helping to reduce perceptions of pain and distress (e.g., Nilsson, 2008; Hsieh et al., 2014; Hole et al., 2015; van der Heijden et al., 2015; Lee, 2016; Garza-Villareal et al., 2017; Kühlmann et al., 2018; Martin-Saavedra et al., 2018b; Lunde et al., 2019; Chai et al., 2020; Ginsberg et al., 2022; Loewy, 2022; Powers et al., 2022; Ting et al., 2022; Lee et al., 2023). Indeed, the belief that music, whether listening or performing, can alter the human pain experience has a long history, dating back to the ancient Greeks, and its potential healing properties have long been appreciated by indigenous cultures around the world (Thaut, 2015).

The subjective experience of acute or chronic pain is complex and multidimensional, influenced by many intersecting factors (e.g., Melzack and Casey, 1968; Koyama et al., 2005; Linton and Shaw, 2011; Melzack and Katz, 2013; Noel et al., 2015; Fillingim, 2017; Casey, 2023). Inter-individual differences in noxious sensitivity have been identified (Cheng et al., 2015; Tu et al., 2019; Zou et al., 2021; Powers et al., 2022), which likely include differences within and between “pain-free” subjects participating in trials and those experiencing chronic pain. Thus, it is to be expected that the impact of music on the pain experience may vary across people, the type of noxious stimulus and experimental situations (Chai et al., 2020; Irons et al., 2020; Cabon et al., 2021; Sihvonen et al., 2022; Ting et al., 2022). Furthermore, there may be limited change in the perception of pain intensity, yet reduced subjective feelings of unpleasantness (e.g., Lu et al., 2019; Antioch et al., 2020; Powers et al., 2022).

In addition to such differences, there is considerable heterogeneity in study design and analysis when studying the impact of music-based interventions on the perception of acute or chronic pain. Variability encompasses:

- whether the music is part of a designed music therapy program, which can involve not only listening but active participation, composition or improvization (Hauck et al., 2013; Metzner et al., 2022; Schneider et al., 2022), whether the music is selected and administered by a clinician, or whether it is chosen by the individual who is experiencing pain.
- when listening to music: the type of music that is presented, when and for what period(s) of time, whether it is live or pre-recorded, and the presence or absence of lyrics (e.g., Sakamoto et al., 2018; Martin-Saavedra et al., 2018b; Richard-Lalonde et al., 2020; Wu et al., 2021; Loewy, 2022; Chow et al., 2023; Lee et al., 2023). When listening to favorite music chosen by a patient, pleasant and relaxing choices are often shown to be the most effective (Nilsson et al., 2005; Nilsson, 2008; Roy et al., 2008; Dobek et al., 2014; Hsieh et al., 2014; Garza-Villareal et al., 2017; Lin et al., 2019; Lunde et al., 2019; Antioch et al., 2020; Howlin and Rooney, 2021; Howlin et al., 2022; Colebaugh et al., 2023; Lu et al., 2023; Valevicius et al., 2023).
- the extent to which music’s impact can be distinguished from non-specific effects (Klassen et al., 2008; Koelsch et al., 2011; Kühlmann et al., 2018; Lunde et al., 2019, 2022; Itskovich et al., 2022).
- more general influences relating to social context, for example whether listening alone or in the presence of others (Chanda and Levitin, 2013; Linnemann et al., 2016) and the nature of the interaction between a patient and the clinician/researcher (Kucerova et al., 2023).

Despite these many differences in study design, significant beneficial effects of exposure to music on the experience of pain have consistently been documented in adults, neonates and children (e.g., Klassen et al., 2008; van der Heijden et al., 2015; Johnson et al., 2021; Ting et al., 2022), and across a range of acute and chronic conditions (Garza-Villareal et al., 2017). For example in childbirth (e.g., Simavli et al., 2014; Maleki and Youseflu, 2023), dysmenorrhea (Martin-Saavedra and Ruiz-Sternberg, 2018), laparoscopy (Choi et al., 2023), orthopaedics (Lin et al., 2019; Yu et al., 2020), cancer (Chow et al., 2023; Huang and Huang, 2023), fibromyalgia (Guétin et al., 2012; Usui et al., 2020), dermatology (Riew et al., 2023) and pre-and post-operative care during cardiovascular (Voss et al., 2004; Nilsson, 2009; Wang et al., 2020; Chandrababu et al., 2021; Kakar et al., 2021) or abdominal surgery (e.g., Good et al., 2005; Dale, 2021). There is evidence of music providing pain relief in patients being treated for burns (Whitehead-Pleaux et al., 2007; Hsu et al., 2016; Wu et al., 2021), and for patients in emergency departments or intensive care units (Richard-Lalonde et al., 2020; See et al., 2023; Vijay and Hauser, 2023). In most of these examples the amelioration of aspects of the pain experience - especially any accompanying negative affect such as stress or anxiety – are also reduced, indicative of the multifactorial influence of music on human psychophysiology.

In this review we consider the relevant role (s) of neurophysiological, neurochemical, humoral, and immunological factors, and the possible sites of action – in the periphery, spinal cord, brainstem, limbic system, and cerebral cortex. We discuss the extent to which these factors, individually or in combination, underpin the impact that music has on the quality, intensity and emotional/affective aspects of acute and chronic pain. In so doing, we acknowledge there remains controversy about the respective roles that diverse central and peripheral/systemic processes play in these experiences. This is complex because for each person the musical experience intersects with many psychophysiological elements linked to pain perception including anxiety, threat, distraction, expectation and anticipation, relaxation, positive valence, reward as well as developmental experiences.

### What is pain?

In 2020, The International Association for the Study of Pain (IASP) approved the following (updated) definition of pain: An unpleasant sensory and emotional experience associated with, or resembling that associated with, actual or potential tissue damage (Raja et al., 2020). This revised definition, arrived at after much lively consultation, took into account recent advances in our understanding of pain, and emphasized amongst other things that pain is a personal experience and that pain and nociception are not synonymous. As summarized by Sluka and George (2021), “These advances include knowledge of the neuroplastic nature of pain across the peripheral and central nervous system, relationship of pain with psychosocial factors, advances in pain assessment beyond verbal descriptors, and (the) impact of pain on the individual” (Sluka and George, 2021, p. 2).

The many issues and controversies considered by the IASP when updating the definition reflect the complex nature of pain neurobiology: “Pain can range widely in intensity, quality, and duration and has diverse pathophysiologic mechanisms and meanings. Therefore, defining the concept of pain in a concise and precise manner presents a challenge” (Raja et al., 2020, pg 3). As these authors discuss, during the IASP review process, additional definitions were suggested that emphasized mind–body interactions, cognitive, emotional, anxiolytic and social factors, catastrophization and the apprehension of threat, and of course differences in acute versus chronic pain processing and ongoing perception. These suggestions reflect – to varying extents – the highly influential multidimensional model of pain (Melzack and Casey, 1968), in which interacting sensory-discriminative (intensity, location, quality, duration), affective-motivational (unpleasantness and flight response), and cognitive-evaluative (appraisal, cultural values, context, cognitive state) components subserve the pain experience.

A recent review provides an update on the current status of the multidimensional model of pain neurobiology, emphasizing the perceptual fusion resulting from co-activation of “physiologically and anatomically distinct but interacting CNS structures each separately mediating sensory discriminative, affective, and cognitive aspects of pain” (pg 1, Casey, 2023). Summatively, there is no distinct substrate in the human brain that subserves pain (e.g., Leknes and Tracey, 2008; Boll et al., 2018; Knudsen et al., 2018; Martucci and Mackey, 2018; Bannister, 2019; Esposito et al., 2019; Usui et al., 2020; Gamal-Eltrabily et al., 2021; Mercer Lindsay et al., 2021; Tan and Kuner, 2021; Zhang et al., 2022; Neugebauer and Kiritoshi, 2023). Rather, interconnected co-active elements manifest jointly in the conscious experience of pain. These include afferent nociceptive input, involvement of periaqueductal grey (PAG), rostral ventromedial medulla (RVM) and noradrenergic brainstem nuclei in descending modulation of these inputs, processing in the ventroposterior, intralaminar and paraventricular thalamus, somatosensory cortices, and complex patterns of activity in limbic system pathways [hypothalamus, septal nuclei, amygdala, anterior cingulate cortex (ACC) and hippocampus], the insula, nucleus accumbens (NAc) and regions in frontal cortex (dorsolateral and mediolateral prefrontal cortex, orbitofrontal cortex), as well as the movement system (motor and premotor cortex, basal ganglia and cerebellum) (e.g., Gombaut and Holmes, 2022; Li et al., 2024). The physiological mechanisms underpinning non-specific effects (classically, placebo, and nocebo effects) also feature in the functional substrates for pain experiences (Tracey, 2010; Lunde et al., 2019; Jinich-Diamant et al., 2020; Kummer et al., 2020; Tan and Kuner, 2021; Itskovich et al., 2022). Together, although strict anatomic specificity does not appear to exist, there is “mounting evidence for functional segregation of distinct aspects of sensory coding, intensity coding, aversion and negative affect across neocortical domains and specific pathways, with important differences between acute and chronic pain and between inflammatory and neuropathic pain” (Tan and Kuner, 2021, p. 468). The latter pain experiences have a complex pathophysiology and generally result from injury or disease in either central (e.g., post-stroke pain) or peripheral (e.g., nerve damage, post-viral or diabetic neuropathy) parts of the nervous system (Baron et al., 2010).

**Figure 1:**
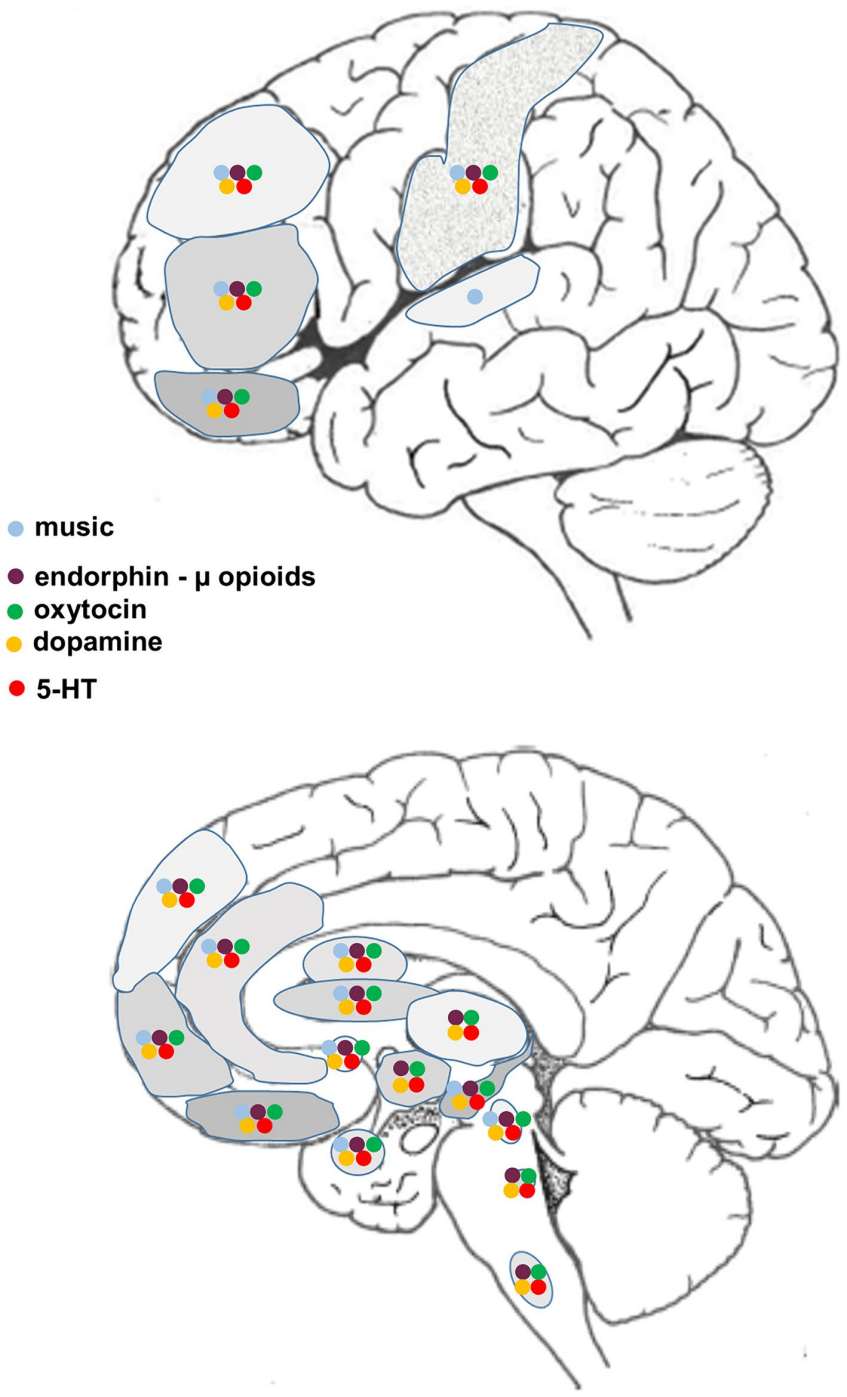
Diagrammatic representation of supraspinal regions involved in the processing of music and/or pain, and the associated distribution of relevant neurotransmitters/neuromodulators.

The neural circuitry associated with the reception and production of music has been extensively documented, much of it obtained using functional magnetic resonance imaging (fMRI). Neural networks that are associated with human musicality are widespread, beginning in the peripheral auditory apparatus (cochlea) and projecting through various brainstem nuclei to the auditory midbrain (inferior colliculus) and thence to the auditory cortex in the temporal lobe via the medial thalamic geniculate nucleus. Different regions within the cerebral cortex – often with a bias towards the right hemisphere – encode different aspects of music such as pitch, rhythm, timbre, melodic contour and so on. However, in the context of the current discussion, of particular relevance are those music-responsive regions known to be involved in emotion (positive or negative), resilience, reward, motivation, expectation and the facilitation of positive social interactions and memories (e.g., Menon and Levitin, 2005; Sharot et al., 2007; Zatorre and Salimpoor, 2013; Koelsch, 2014; Hopper et al., 2016; Harvey, 2017, 2020; Chen Y. L. et al., 2022; Chen W. G. et al., 2022; Vuust et al., 2022) (Figure 1).

In the context of music-induced analgesia, distinctions between acute and chronic pain, and between inflammatory and neuropathic pain, are particularly important. First because different mechanisms and circuits are involved in these different experiences of pain, and second because many of the preclinical studies that examine the impact of music on pain perception assess responses to acute noxious stimuli in pain-free subjects. The relevance of such observations to chronic pain requires some caution given the established changes in plasticity, neurochemistry, connectivity, and excitatory/inhibitory balance that have been documented in the nervous systems of people with chronic pain (Apkarian et al., 2004; Robinson et al., 2011; Pertovaara, 2013; Zhao et al., 2017; Martucci and Mackey, 2018; Nascimento et al., 2018; Esposito et al., 2019; McCarberg and Peppin, 2019; Kummer et al., 2020; Usui et al., 2020; Tan and Kuner, 2021; Higginbotham et al., 2022; Zhang et al., 2022; Hao et al., 2023; Labrakakis, 2023; Yan et al., 2023; Li et al., 2024). Different neurotransmitters/neuromodulators may act to reduce pain in acute situations but enhance pain and discomfort in chronic conditions. There are also important changes in inflammatory and immunomodulatory systems in both the periphery and centrally that mediate chronic pain states, including hyperalgesia and allodynia (e.g., Luchting et al., 2015; Ronchetti et al., 2017; Sawicki et al., 2021; Chen Y. L. et al., 2022; Chen W. G. et al., 2022 Seidel et al., 2022).

There are now many comprehensive reviews on the complex processing and perception of pain, and it is not our intention to review this literature again. Our aim is to focus on those structural, molecular and functional aspects of acute and chronic pain processing that reveal a close association with what is currently known about the neurobiology and neurochemistry of human musicality (Figure 1). We will also briefly address likely interactions between music, relaxation and meditation (Davis and Thaut, 1989; Nascimento et al., 2018; Bellosta-Batalla et al., 2020; Wipplinger et al., 2023), and consider whether the validity of self-reported pain intensity and unpleasantness typical of many clinical trials may sometimes be confounded by the prosocial, cooperative and trust building effects that familiar, well-loved, self-chosen music can have on human empathic behavior (e.g., Freeman, 2000; Pearce et al., 2015; Harvey, 2017, 2020; Savage et al., 2021; Itskovich et al., 2022). Note also that an individual’s positive or negative expectancy can influence the relative efficacy of pain relief (Koyama et al., 2005; Atlas et al., 2010; Tu et al., 2019; Boselie and Peters, 2023; Koppel et al., 2023) and may even lead to unwillingness to engage in a pain-related trial if outcomes are predicted to be negative or unpleasant (Karos et al., 2018; Ji and MacLeod, 2023). In sum, we hope to provide further insight into the mechanisms that underlie music’s impact on pain perception, which should aid in developing more effective synergistic approaches if music-related therapies are combined with multifaceted clinical treatments used for the relief of acute or chronic pain, and reduction of associated anxiety and stress.

### Neurochemistry: a nexus for musical modulation of pain

From a structural point of view, there are numerous cortical and subcortical areas in which neural activity is altered when processing aspects of pain that are also active when interrogated using music-related stimuli. These regional associations provide potential sites for music-pain interactions, likely mediated via a complex interplay between various neuromodulatory systems (Figure 1). Systems known to be affected by musical activities (Chanda and Levitin, 2013) and likely of relevance to complex pain perception are also highlighted:

- β-endorphin and receptors (purple) (Dunbar et al., 2012; Tarr et al., 2015; Weinstein et al., 2016; Tarr et al., 2017)
- oxytocin and receptors (green) (Grape et al., 2003; Kreutz, 2014; Keeler et al., 2015; Yuhi et al., 2017; Harvey, 2020; Good and Russo, 2022)
- dopamine and receptors (yellow) (Blood and Zatorre, 2001; Zatorre and Salimpoor, 2013; Ferreri et al., 2019; Sihvonen et al., 2022)
- serotonin and receptors (red) (Evers and Suhr, 2000; Barrett et al., 2018; Park et al., 2023).

We have included serotonin here because, although the evidence for links between this monoamine and music are at present less clearcut, its role in the modulation of pain is well-established (see below) and its influence on mood (e.g., depression) is important in influencing the experience of pain (e.g., Hao et al., 2023; Cui et al., 2024). In addition to the neuromodulators mentioned above, many more have been implicated in processing and modifying the perception of pain, acute or chronic, centrally and/or peripherally (Yam et al., 2018; Gamal-Eltrabily et al., 2021). These include noradrenaline, the excitatory neurotransmitter glutamate and inhibitory neurotransmitter gamma-aminobutyric acid (GABA), nitric oxide, cannabinoids, histamine, prostaglandins, and a range of pro-inflammatory agents (cytokines and tachykinins).

The great majority of these neuromodulators do not necessarily act independently of each other. They interact in multiple ways at the synaptic and receptor level, with the potential to affect numerous intracellular signaling pathways. At the synaptic level there can be interactions both pre-and post-synaptically, the nature of these interactions varying dependent on the precise synaptic arrangements and circuits that exist in different parts of the CNS. At the receptor level, many of the receptors for neurotransmitters/neuromodulators that have at least some influence on pain perception are G protein coupled receptors (GPCRs). These are dynamically regulated (Cahill et al., 2007) cell surface receptors that are coupled to so-called G proteins that regulate diverse cellular functions. Different GPCRs can come together to form complexes that influence their functionality, resulting in altered down-stream intracellular signaling (Borroto-Escuela et al., 2017, 2022; Chruścicka et al., 2019; Hernández-Mondragón et al., 2023). From a music and pain perspective, potentially relevant GPCR complexes include dopamine and serotonin receptors, oxytocin and serotonin receptors, oxytocin and dopamine receptors, and dopamine and glutamate receptors. Finally, there can be allosteric modulation of GPCRs in which one ligand binds to a secondary binding site on a receptor thereby enhancing or diminishing the efficacy of the primary ligand for that particular receptor (Foster and Conn, 2017).

In the following discussion the aim is to introduce some of the neuromodulating agents whose impact seem most relevant in acute and chronic pain relief (Figure 1). This is a vast literature, thus it has proved necessary in many cases to cite recent reviews rather than original reports. The exception is a closer focus on oxytocin and its potential role in music-induced analgesia. Recent studies in animal models have revealed details of oxytocinergic circuitry (Son et al., 2022) and the role of this peptide in pain sensitivity (Boll et al., 2018; Li et al., 2021). The multifactorial nature of oxytocinergic relief in acute and chronic pain, and the ameliorative psychological and physiological effects of this peptide on nociception are increasingly being appreciated in the clinical pain field (e.g., Tracy et al., 2015; Goodin et al., 2015; Paloyelis et al., 2016; Poisbeau et al., 2018; Schneider et al., 2020; Mekhael et al., 2023). However, relatively few systematic analyses on music-based interventions consider oxytocin in the context of pain relief, even though links between this peptide and human musicality are evident (Harvey, 2020). While most of the analgesic effects of music are mediated by complex pathways and interactions within the CNS there is evidence for additional effects that are mediated in the periphery.

#### Serotonin

Serotonin (or 5-hydroxytryptamine) is a monoamine transmitter produced in the raphe nuclei in the brainstem, exerting widespread influence in the CNS. Serotonergic pathways have long been known to be involved in modulating acute and chronic pain, anxiety and depression, diverse and complex effects that are mediated by a range of receptor subtypes (Bardin, 2011; Yam et al., 2018; Heijmans et al., 2021; Hao et al., 2023). The central actions of this monoamine can be either inhibitory (analgesia) or excitatory (hyperalgesia), and it has pro-inflammatory effects in the periphery. For example, serotonergic neurons project inferiorly from the raphe nuclei to the segmental dorsal horn, with either facilitatory or inhibitory effects on local neurons (Bear et al., 2016). These projections are a component of the endogenous diffuse noxious inhibitory control (DNIC) system. The DNIC system is a functional grouping of hypothalamic and ponto-medullary neurons (the PAG, raphe nuclei and locus coeruleus) that modulate signaling at spinal levels and are themselves subject to influence from forebrain and limbic regions, and autonomic and somatosensory systems. DNIC operates through opioid peptides and nor-adrenaline as well as serotonin (Baldry and Thompson, 2005). The end-result is prolonged altered activity in primary and projection neurons within the dorsal and anterolateral tracts (Zain and Bonin, 2019; Pereira-Silva et al., 2023). Notable too are the systemic central influences of serotonergic raphe neurons projecting rostrally to higher centers (Bear et al., 2016).

#### Noradrenaline (norepinephrine)

Noradrenaline is a catecholamine produced in several small brainstem nuclei in the pons and medulla. Descending projections from the locus coeruleus (A6) and perhaps A5 are noradrenergic contributions to DNIC, with antinociceptive effects (e.g., Kucharczyk et al., 2023). However, typical of the complex phenomenae of pain and responses to injury, peripheral noradrenaline has little impact in pain-free individuals yet there is evidence for both anti and pro-nociceptive effects in injured or inflamed tissues (Pertovaara, 2013). Moreover, circulating nor-adrenaline (and adrenaline) is critical to the hormonal stress response, with distributed effects contributing to other tangible aspects of the experience of pain or music; notably, respiratory and cardiac dynamics, blood pressure, and metabolism (Bear et al., 2016).

#### Dopamine

As with serotonin, the dopaminergic system is a widely distributed network with potential influence on pain perception. Dopaminergic neurons from the substantia nigra, ventral tegmental area, and subthalamic nuclei modulate functions involving prefrontal and cingulate cortices, the basal ganglia, NAc, thalamus, hippocampus and brainstem (Figure 1, Bear et al., 2016). Learning, reward and movement execution are major implications from cortical dopaminergic transmission. Subcortically, dopamine influences PAG function that, together with opioid signaling, contributes to descending modulation of nociception, outcomes that vary depending on the type of dopamine receptor that is involved (Li et al., 2019; Kummer et al., 2020; Cabon et al., 2021; Neugebauer et al., 2023). There are direct projections from the (hypothalamic) A11 nucleus to the spinal trigeminal nucleus in the brainstem and dorsal horn of the spinal cord, with likely modulatory effects on nociception (Charbit et al., 2009; Li et al., 2019). Chronic pain experiences are associated with significant changes in functional connectivity, including evidence of altered dopamine signaling in for example striatum, PFC and NAc (Jaaskelainen et al., 2001; Li et al., 2019; Kummer et al., 2020; Zhang et al., 2022). Leknes and Tracey (2008) cite observations linking altered dopamine related activity with changes in pain perception and the experience of pleasure, likely via interactions with opioids (see below). Indeed, they argue that: “These two neurotransmitter systems are thus likely to mediate the brain’s common currency, allowing for action selection based on the comparison between competing pleasant and aversive events” (Leknes and Tracey, 2008, p. 318).

#### Opioids

The body makes its own opioid peptides (e.g., β-endorphin, enkephalins, and dynorphin) that bind to different opioid receptors. β-endorphin signals through μ-opioid receptors, enkephalins bind to both μ-and δ-receptors, the latter with higher affinity, and dynorphin binds to κ-receptors. The μ-and δ-opioid receptors have similar functions but in regions such as ACC, PFC, amygdala and NAc the κ-opioid receptor can have an opposing influence, and there is also interaction with dopaminergic circuits (Neugebauer et al., 2023). Many studies have focused on β-endorphin and the distribution of μ-opioid receptors. β-endorphin is produced mostly in hypothalamic and pituitary cells, and released into the bloodstream from the pituitary gland as well as influencing many regions within the CNS. In the brain the μ-opioid receptor is widely distributed and found in high density in ACC, PFC, OFC, insula, NAc and somatosensory cortex, as well as in the amygdala, hippocampus, diencephalon, PAG, locus coeruleus, RVM and spinal cord (Figure 1). In humans, μ-opioid receptor availability is generally slightly higher on the right hand side of the brain, with the exception of NAc and amygdala, where a left sided bias is seen (Kantonen et al., 2020). Note that peripheral levels of β-endorphin do not necessarily predict how a given individual responds to pain or opioid analgesia (Leonard et al., 1993; Bruehl et al., 2017), perhaps related to inter-individual variability of μ-opioid receptor levels as well as age-related changes in receptor availability (Kantonen et al., 2020).

Opioid medicines are widely used to treat pain but have substantial issues (Deyo et al., 2015; de Sola et al., 2020). Because opioid receptors are distributed throughout the CNS and periphery, notably the gastrointestinal tract, these medicines have multiple adverse effects (e.g., constipation), lose effectiveness with prolonged use and are potentially addictive (Deyo et al., 2015; Cashin et al., 2023). In some instances there is development of increased pain - a secondary condition termed opioid-induced hyperalgesia (Higginbotham et al., 2022). In addition, as noted earlier, opioids can influence the activity of other receptor types including dopamine, serotonin, oxytocin, glutamate and GABA – influences that are often reciprocal. Clinical examples of these interactions include the opioid medicines tramadol (serotonergic and noradrenergic [Grond and Sablotzki, 2004]) and tapentadol (noradrenergic, weakly serotonergic [Singh et al., 2013]). As a result, opioids can influence not only nociception at a number of levels but also many other psychophysiological and cognitive functions including learning and memory, reward, social interactions, mood and stress (Pilozzi et al., 2021; Putnam and Chang, 2022; Neugebauer et al., 2023). For all of these reasons, considerable effort is directed towards reducing opioid usage, an outcome that – as discussed later - can result from the use of music-based interventions in pain management.

In human positron emission tomography (PET) studies, use of radioactive ligands has revealed alterations in the binding potential of μ-opioid receptors in chronic pain sufferers (e.g., Zubieta et al., 2001; Maarrawi et al., 2007; Li Y.- J. et al., 2023; Li Z. et al., 2023). Depending on the type of chronic pain under examination, a decrease in μ-opioid receptor binding has been described in OFC, PFC, ACC, NAc, striatum, ventral tegmental area (VTA), PAG, insula and medial thalamus. This is generally interpreted as an indication of increased receptor occupancy and therefore greater release and presence of endogenous opioids. However, it is possible that these changes may also reflect an altered affinity of a particular ligand for μ-opioid receptors and/or receptor down regulation (Niikura et al., 2010). Importantly, combined with the release from axons there is more distant and distributed volume transmission of β-endorphin in the CSF and brain, broadening and extending its influence on function (Veening et al., 2012).

In the periphery, β-endorphin tends to reduce immune responses, however opioids can also reduce physiological responses to stress, and have anti-inflammatory actions (Pilozzi et al., 2021). In this context, peripheral opioid expression levels are altered in chronic pain states and have been reported to be inversely related to the degree of perceived pain (Brejchova et al., 2021). In addition, there is *ex vivo* evidence that all three opioid receptors are expressed by circulating immune cells (macrophages, monocytes, lymphocytes and granulocytes) themselves, and when stimulated these cells can release opioid peptides (β-endorphin, met-enkephalin and dynorphin) that act on receptors expressed in DRG and trigeminal ganglion neurons, resulting in reduced nociceptive transmission (Sun et al., 2019; Machelska and Celik, 2020; Brejchova et al., 2021).

#### Oxytocin

In the brain this nine amino-acid peptide is synthesized in several circumscribed regions within the hypothalamus. It is released into the bloodstream from the posterior pituitary but oxytocin expressing neurons also send axons to numerous parts of the CNS. Brain regions showing overlap of oxytocin receptors with areas activated by music that is familiar, rewarding, arousing and/or motivating are summarized in Figure 1 (Quintana et al., 2019; Harvey, 2020). Oxytocin has a remarkably wide range of actions both peripherally and centrally. It is perhaps best known for its role before and after childbirth, stimulating the uterus and lactation. It is also important in attachment and bonding, and together with the opioids is linked to many aspects of human social behavior including cooperativity, trust and emotional empathy (Harvey, 2020; Putnam and Chang, 2022). The peptide also has beneficial systemic effects on the cardiovascular and immune/inflammatory systems, and the regulation of appetite and glucose homeostasis (e.g., Li et al., 2017; Lawson et al., 2019; Onaka and Takayanagi, 2019; Reiss et al., 2019; Jankowski et al., 2020; Niu et al., 2021; Liu et al., 2022; Nishimura et al., 2022). The immune regulatory and anti-inflammatory effects may have an impact on pain perception, although in humans there is evidence for altered oxytocin levels in some chronic pain conditions (Goodin et al., 2015; Mekhael et al., 2023). Importantly, oxytocin is now well established to act centrally, both in the spinal cord and brain, to reduce the perception of pain (Yang et al., 2022). These anti-nociceptive actions may be mediated by the oxytocin receptor itself or via interaction with opioid and other neuromodulator systems, but in addition the peptide has calming and anxiolytic effects (Kirsch et al., 2005; Neumann and Slattery, 2016; Herpertz et al., 2019), is often negatively correlated with cortisol, and can reduce stress (Lee et al., 2015; Schladt et al., 2017; Neumann and Landgraf, 2019). In some circumstances oxytocin may act to reduce memory of adverse or fearful events (e.g., Donadon et al., 2018; Hu et al., 2019; Giovanna et al., 2020; Baldi et al., 2021), all important in the affective, psychophysiological response to acute and chronic pain (Boll et al., 2018).

The physiological actions of oxytocin are mediated by its own receptor, but oxytocin is also a positive allosteric modulator of μ-and κ-opioid receptors, enhancing signalling when studied *in vitro* (Meguro et al., 2018; Miyano et al., 2021). Consistent with this, oxytocin induced analgesia after injection of the peptide into rat PAG or NAc is blocked by μ-κ- but not δ-opioid receptor blockers (Ge et al., 2002; Gu and Yu, 2007). As discussed earlier, the oxytocin receptor also forms functional complexes with dopamine and serotonin receptors. At the synaptic and pathway level, the actions of oxytocin released from the hypothalamus can interact with and modulate other neurotransmitter systems including dopamine (Li et al., 2020), serotonin (Yoshida et al., 2009; Borroto-Escuela et al., 2022), glutamate, GABA (e.g., Breton et al., 2008; Nishimura et al., 2022), and the important nociceptive receptor transient receptor potential vanilloid 1 (TRPV1) (Nersesyan et al., 2017; Sun et al., 2018; Biurrun Manresa et al., 2021; Shuba, 2021).

In animal studies, analgesic effects of oxytocin have been described after experimentally induced acute (Rash et al., 2014), neuropathic and inflammatory pain experiences (Yu et al., 2003; Condes-Lara et al., 2005; Eliava et al., 2016; Sun et al., 2018; Hilfiger et al., 2020; Li et al., 2021; Esmaeilou et al., 2022; Nishimura et al., 2022; Iwasaki et al., 2023; Krabichler and Reynders, 2023; Li Y.- J. et al., 2023; Li Z. et al., 2023), and increased expression of oxytocin receptors has been reported after chemotherapy-induced neuropathic pain in mice (Li et al., 2023a). Similar to the distribution of oxytocin receptors in the CNS (Insel and Shapiro, 1992; Quintana et al., 2019) the anatomical location of these oxytocinergic effects is widespread, although in mice at least, there is not always congruence between oxytocin output and receptor expression (Son et al., 2022). Thus, while preclinical rat studies have shown that the analgesic effects of oxytocin can be blocked by both oxytocin receptor and sometimes μ-opioid receptor antagonists (e.g., Ge et al., 2002; Miranda-Cardenas et al., 2006; Gu and Yu, 2007), as with β-endorphin, release of oxytocin beyond synaptic sites into the extracellular space and even into the CSF suggests more widespread indirect effects of the peptide, significantly extending its potential modulatory influence and functional range (Chini et al., 2017; Son et al., 2022).

With regard to nociception, oxytocin influences physiological activity in known pain-related sites in the brain such as PAG, prefrontal and somatosensory cortex, ACC, insula, amygdala, NAc, raphe and hypothalamus (Figure 1) (e.g., Gu and Yu, 2007; Eliava et al., 2016; Boll et al., 2018; Poisbeau et al., 2018; Li et al., 2021; Saito et al., 2021; Nishimura et al., 2022; Yang et al., 2022; Iwasaki et al., 2023; Li et al., 2023a). Receptors are also found in the spinal cord and on a proportion of peripheral sensory neurons in the dorsal root ganglia (DRG) (Condes-Lara et al., 2005; Gong et al., 2015; González-Hernández et al., 2017; Poisbeau et al., 2018; Martinez-Lorenzana et al., 2021; Noguri et al., 2022; Yang et al., 2022; Li Y.- J. et al., 2023; Li Z. et al., 2023). Evidence from transgenic rat studies has also been presented suggesting effective analgesic action via both neuronal and humoral pathways (Nishimura et al., 2022). In this experimental model, oxytocin reduced pro-inflammatory mast cell degranulation and, under certain pathological conditions, induced anti-inflammatory effects that were mediated via the hypothalamic/pituitary/adrenal axis. Central analgesia involved activation of neurons in locus coeruleus and dorsal raphe. In turn there was altered inhibition in the dorsal horn which could be specifically blocked using an oxytocin receptor antagonist (Nishimura et al., 2022).

### Mechanisms underlying the psychophysiological effects of music on the experience of pain

As summarized earlier a large number of recent meta-analyses and systematic reviews have documented the extent to which music-based interventions can act as an analgesic and anxiolytic during various types of medical and surgical intervention. The many physiological and imaging studies that attempt to discern exactly how music impacts the experience of acute pain in otherwise healthy subjects are clearly pertinent here. However, for many neurotransmitters/neuromodulators there are altered expression levels, changes in receptor expression, and differential effects (inhibitory or facilitatory) on nociception when assessed in chronic versus acute pain states (e.g., Elman and Borsook, 2016; McCarberg and Peppin, 2019; Tan and Kuner, 2021; Seidel et al., 2022; Zhang et al., 2022). Thus elucidating the likely mechanisms that influence the way music reduces the subjective burden of chronic pain may prove more difficult.

Figure 1 presents a summary of the major CNS regions involved in the neurobiology of acute and chronic pain that have also been implicated in processing various aspects of human musicality, most of the information obtained from fMRI studies. While such regional congruities do not necessarily mean that the same circuits or neuromodulators are involved (Peretz et al., 2015), this comparison does suggest many potential interactive sites where music can ameliorate the perception of pain. Also included are the sites of action of four of the major neurotransmitter/neuromodulator systems known to be affected by music. Clinically, there are few studies using receptor blockers to examine the underlying physiological/neurochemical basis for the effect of music-based interventions on acute and/or chronic pain relief. Some trials report decreased opioid use after music-based therapy (reviewed in Nilsson, 2008; see also Hole et al., 2015; Lee, 2016; Fu et al., 2020; Wu et al., 2021), others have found no change in drug requirements (reviewed in Nilsson, 2008; see also Hsu et al., 2016; Lin et al., 2019; Kakar et al., 2021). In healthy subjects, Lunde et al. (2022) found that administration of either an opioid or dopamine antagonist did not reduce the beneficial analgesic effect of music, although it should be noted that the music was not self-chosen, which may be relevant. We are unaware of any studies using oxytocin-specific antagonists in music therapy trials.

Due to the multifaceted nature of the interactions between human musicality and pain perception, there are a number of different ways this section could be constructed. We have chosen to consider music’s impact by first discussing potential mechanisms in the periphery, then in the sensory ganglia, spinal cord and brainstem, and finally in limbic system and cerebral cortex, cognizant of the fact that these are not independent entities and that, depending on circumstances, some if not all of these levels may play a role in music’s analgesic and anxiolytic properties.

#### Autonomic nervous system

The autonomic nervous system (ANS) regulates the internal physiological environment, including blood pressure, heart rate, digestion, and respiration. It also has a critical role in sexual arousal. CNS influences arise from various regions in the brainstem as well as the limbic system and hypothalamus (Ellis and Thayer, 2010). There are three components – the sympathetic, parasympathetic and enteric nervous systems. Put simply, the sympathetic and parasympathetic nervous systems work together in a homeostatic fashion, often with opposing outcomes. The former tends to drive activity (“fight or flight” response), increase heart rate and blood pressure etc., while the latter acts to reduce heart rate and blood pressure, and increase more rest-related activities such as digestion. Importantly, mental or physical stress is associated with greater sympathetic activity, a serious health risk if long-term stress is not controlled.

A useful measure of sympathetic/parasympathetic balance is heart rate variability (HRV). Lack of variability is associated with stress, anxiety and/or chronic pain, and has been linked to dysregulated parasympathetic activity (Ellis and Thayer, 2010; Tracy et al., 2016; Ginsberg et al., 2022). Although some heterogeneity is evident, analysis of music-related studies that have measured HRV as well respiratory rate and other cardiovascular signs show that music-based interventions increase HRV and can – dependent to some extent on the type of music that is presented (Davis and Thaut, 1989; Knight and Rickard, 2001; Ooishi et al., 2017) - lower heart rate and blood pressure, all indicative of increased vagal tone and enhanced activity in the parasympathetic nervous system (Okada et al., 2009; Koelsch and Jäncke, 2015; Wang et al., 2020; Ginsberg et al., 2022; Metzner et al., 2022; Ting et al., 2022; Cao and Zhang, 2023; Park et al., 2023). These signs of re-balancing of the ANS are often associated with reduced pain perception and often accompanied by a reduction in the stress related hormone cortisol (McKinney, Antoni, et al., 1997a; Grape et al., 2003; Kreutz et al., 2004; Koelsch et al., 2011, 2016; Ooishi et al., 2017; Fu et al., 2019). Reduced plasma levels of adrenaline and nor-adrenaline have also been reported (Okada et al., 2009). While not directly linked to peripheral nociceptive processing, these indications of reduced sympathetic activity and stress are linked to changes in activity in frontal and prefrontal cortex and are important when considering the impact that anxiety can have, increasing the perception of chronic pain (Du et al., 2022).

#### Inflammation and immune reactivity

Inflammatory and immunomodulatory changes contribute to the development of, and prolong the experience of, chronic pain (Ronchetti et al., 2017; Sawicki et al., 2021; Seidel et al., 2022). Peripheral inflammation or tissue injury involves an increased presence of mast cells, lymphocytes, monocytes and macrophages, with greater levels of pro-inflammatory agents such as IL-6 and IL-1β, tumor necrosis factor (TNF) α, bradykinin, prostaglandin-2, serotonin histamines, tachykinins, substance P, and interferon-γ (Luchting et al., 2015; Ronchetti et al., 2017; Yam et al., 2018; Seidel et al., 2022). These agents promote inflammation and healing (in early stages), alter (“sensitize”) response profiles of sensory afferents and feature in systemic processes subserved by the HPA axis. Throughout the CNS, glial cell activity changes, particularly microglia and astrocytes (Sawicki et al., 2021; Chen Y. L. et al., 2022; Chen W. G. et al., 2022), potentially affect the efficacy of opioids (Pahan and Xie, 2023) and alter synaptic dynamics.

With regard to music, its analgesic and immuno-homeostatic effects have been linked to several neuromodulatory systems (Fancourt et al., 2014). For example:

- Listening to music (Charnetski et al., 1998) or participation in choral singing (Beck et al., 2000; Kreutz et al., 2004) significantly increased salivary levels of immunoglobulin-A, an antibody important for immune function.
- Beck et al. (2000) also found reduced levels of cortisol (see also Khalfa et al., 2003).
- Music-based interventions were shown to reduce plasma levels of the pro-inflammatory cytokine IL-6 (Stefano et al., 2004; Conrad et al., 2007; Okada et al., 2009; Koelsch et al., 2016).

Reduced sensitivity to pain after communal music making has been interpreted as an indication of increased endorphin expression (Dunbar et al., 2012; Tarr et al., 2015; Weinstein et al., 2016). Consistent with this, the opioid antagonist naltrexone blocked the decrease in pain sensitivity reported after synchronous dancing (Tarr et al., 2017). These are interesting observations, however the perception of pain may not always reflect circulating levels of β-endorphin (Bruehl et al., 2017; Ahn et al., 2019), and there is inconsistency in music-induced changes in plasma levels of factors related to β-endorphin activity (McKinney et al., 1997b; Stefano et al., 2004). Increased serum (Grape et al., 2003; Nilsson, 2009; Keeler et al., 2015) or salivary (Kreutz, 2014; Yuhi et al., 2017) levels of oxytocin have been reported after singing or group drumming, as well as when listening to relaxing music (Ooishi et al., 2017). These effects can be moderated by social context, mood and level of empathy (Schladt et al., 2017; Eerola et al., 2021; Bowling et al., 2022), changes in the functionality of these systems in various chronic pain states (Bruehl et al., 2012; Ahn et al., 2019; Brejchova et al., 2021; Mekhael et al., 2023), and opioid-oxytocin and opioid-immune interactions (Meguro et al., 2018; Miyano et al., 2021; Pilozzi et al., 2021).

#### Sensory ganglia and spinal trigeminal nucleus

Nociceptors are primary afferents that respond to thermal (heat or cold, burns), mechanical (tissue injury, pressure) or chemical stimuli (ischaemia, inflammation) through a variety of receptors. Response profiles are often higher than other afferents, meaning these neurons transduce stimuli more likely to be threatening or noxious (hence, nociceptors). Nociceptors comprise myelinated Aσ-fibers and unmyelinated C fibers, both of which synapse in select laminae of the dorsal horn or in brainstem nuclei. Their cell bodies are located in the DRG or spinal trigeminal ganglia (Bear et al., 2016).

Phenotypic and functional changes in nociceptors are associated with nascent and persistent pain experiences; often involved in inflammation and immune dysregulation (Esposito et al., 2019). It is possible some aspect of music-induced analgesia may be mediated at this level, principally because nociceptor sub-populations express receptors for β-endorphin, oxytocin, nor-adrenaline and/or serotonin (Gold et al., 1997; Matsushita et al., 2018; Martinello et al., 2022; Noguri et al., 2022). As noted immediately above, music reduces nor-adrenaline, increases oxytocin and has variable effects on serotonin. It is also possible the alterations in the immuno-inflammatory cascade promoted by music positively influence response profiles of nociceptive afferents, given the increased responsiveness of these cells is partly driven by pro-inflammatory signaling.

#### Spinal cord and brainstem

The spinal cord is a major site of nociceptive modulation, involving many neuroactive agents and mediated by complex synaptic interactions between afferent sensory inputs and descending and local excitatory and inhibitory circuits. The functional output from this milieu is further complicated by altered activity and signaling across pain conditions, and by individual differences in response profiles (e.g., D’Mello and Dickenson, 2008; Heijmans et al., 2021; Koning et al., 2023). Recent studies also implicate astrocytes in modulatory mechanisms within spinal cord (Chen Y. L. et al., 2022; Chen W. G. et al., 2022). Overall, in persistent pain conditions there is generally increased ascending nociceptive activity and reduced descending anti-nociceptive influence, the literature not always in agreement as to the extent to which this sensitization is peripheral or central in origin – or perhaps a variable combination of both (e.g., Cheng et al., 2015; Tracy et al., 2015; Bannister, 2019; Brazenor et al., 2022; Mendell, 2022; Treede, 2022; Adams et al., 2023).

Musical modulation of nociceptive transmission at the spinal cord level, potentially involving β-endorphin, oxytocin, dopamine, nor-adrenaline and/or serotonin, has been demonstrated. Listening to favorite music affected neural responses not only in cortex and brainstem but also in the dorsal gray of the spinal cord, interpreted as being due to descending influences originating in the PAG (Dobek et al., 2014). Similarly, the nociceptive spinal reflex, skin conductance and pain perception were reduced after listening to pleasant (but not arousing) music (Roy et al. (2012)). Several recent studies have assessed the effect of music on TSP and CPM/DNIC. While one study did not observe changes in either pain sensitivity mechanism when healthy subjects listened to favorite music (Colebaugh et al., 2023), two other studies reported that TSP but not CPM/DNIC was significantly reduced when listening to music of some kind (Chai et al., 2020; Cabon et al., 2021).

#### Higher centers – cerebral cortex, limbic system, and basal ganglia

The foregoing discussion has provided evidence that at least some of the known antinociceptive impact of music results from physiological effects mediated in the periphery and at spinal and brainstem levels, involving both ascending and descending systems, and likely involving a variable combination of β-endorphin, oxytocin, dopamine, nor-adrenaline and/or serotonin interactions. The potential complexity already evident becomes even greater when considering how exposure to and/or involvement in music influences higher emotional and cognitive processes, and the extent to which these processes influence the overall subjective, multidimensional experience of acute and chronic pain, including descending influence on TSP and DNIC (Dobek et al., 2014; Garza-Villareal et al., 2017; Powers et al., 2022). Elucidating the connectional architecture and underlying mechanisms that directly link music to pain perception at higher levels is not easy because additional contextual influences such as valence, distraction, meditation, fear, anticipation and expectation come into play (e.g., Lunde et al., 2019; McCarberg and Peppin, 2019; Gamal-Eltrabily et al., 2021; Tan and Kuner, 2021; Brazenor et al., 2022; Lunde et al., 2022; Powers et al., 2022; Adams et al., 2023).

As described earlier, in music-based interventions that target subjective pain relief, music is most effective if it is chosen by the person with pain and combines elements that are familiar, pleasant, rewarding and/or anxiolytic. The more focus, engagement, and cognitive agency (Howlin and Rooney, 2021; Howlin et al., 2022, 2023) the person has, the better. Social context also appears to be important (Linnemann et al., 2016). The higher order regions involved in these aspects of a musical experience include the amygdala, ACC, insula, the caudate nucleus and NAc, superior temporal gyrus, somatosensory cortex, and orbitofrontal, ventromedial, dorsomedial and dorsolateral prefrontal cortices (Figure 1). Again note that some, or all, of the major neuromodulator systems in the forebrain known to be influenced by music - β-endorphin, oxytocin, and dopamine – have the potential to be involved.

Several studies have argued that the therapeutic effects of music are not merely due to distraction (Roy et al., 2008; Lu et al., 2019, 2023). Furthermore, music still reduces outcome measurements of pain even when the music is delivered during general anesthesia and therefore cannot be attributed to distractive influence (Nilsson et al., 2003; Simcock et al., 2008; Hole et al., 2015; Kühlmann et al., 2018). On the other hand, Lunde et al. (2022) reported that the analgesic effect of 5 min of (not self-chosen) music in healthy subjects was not blocked by opioid or dopamine antagonists, but the effectiveness of music was predicted by an individual’s expectation that they would obtain pain relief. Others have reported that positive expectancy is less effective than music in decreasing pain sensitivity (Hsieh et al., 2014). The lack of effect of the dopamine antagonist (Lunde et al., 2022) may be due to the type and mode of presentation of the music excerpt given the known role of dopamine in the anticipation and rewarding experience of familiar music (Pereira et al., 2011; Salimpoor et al., 2011; Zatorre and Salimpoor, 2013; Ferreri et al., 2019). Note also that the opioid antagonist naloxone blocked the synchronous dance induced increase in pain threshold although no change in social closeness was registered (Tarr et al., 2017). The likely role of oxytocin and its cognate receptor in communal music making and pain relief (Kenny and Faunce, 2004; Hopper et al., 2016; Weinstein et al., 2016; Harvey, 2020; Good and Russo, 2022) is yet to be examined. Related to these issues, some have found no change in ‘chills’ or subjective responses using opioid blockers when listening to music (Laeng et al., 2021; Mas-Herrero et al., 2023) while others reported a decrease in pleasure after naltrexone delivery (Mallik et al., 2017). Catastrophization and the negative prediction of pain is linked to altered activity in regions such as ACC, prefrontal cortex, cingulate and insula (Tu et al., 2019; Antioch et al., 2020; Koppel et al., 2023), and is associated with an increased nociceptive experience (e.g., Brazenor et al., 2022). Importantly, this type of negative mindset reduces the effectiveness of music-based therapies (Chai et al., 2020; Powers et al., 2022).

The complex web of interactive circuits that underpin the psychophysiological complexities of pain are now well-known, involving multiple regions in cortex, limbic pathways, thalamus, hypothalamus and specific regions in the midbrain and brainstem. An implication of previous sections of this review is that music may contribute to remedial plasticity in circuits and networks altered in the persistent pain experience. Here we discuss another possible mode of interaction between the perception of pain and the experience of music – neural oscillations or brain waves. These rhythmic patterns of synchronized neural activity flowing within and across, and linking, neighboring regions of the brain have different repetitive frequencies (delta, 1–4 Hz; theta, 4–8 Hz; alpha, 8–13 Hz; beta, 13–30 Hz, gamma, 30–120 Hz), each associated with different brain states. Interestingly, there is evidence that oscillatory brain activity is linked to pain perception (Ploner and Gross, 2019; Kenefati et al., 2023; Li et al., 2023b). Pain-induced changes in gamma oscillations in somatosensory cortex have been described (Gross et al., 2007), while noxious heat stimulation in healthy subjects was shown to increase gamma wave activity in bilateral mPFC but decrease alpha and beta wave activity in somatosensory cortex (Nickel et al., 2017). Specific patterns of altered brainwave activity involving theta, alpha and gamma oscillations have been recorded in prefrontal areas and anterior cingulate in people with chronic pain (Kenefati et al., 2023). Supporting a link between gamma oscillations and pain perception, variability in amplitude of these oscillations was found to predict inter-individual pain sensitivity (Hu and Iannetti, 2019) and others have reported links between reduced pain perception and modulated delta, beta or gamma band oscillations (Hauck et al., 2013).

Different frequencies of sound vibration can affect a wide range of neurological and physiological functions, gamma waves (around 40 Hz) having significant effects on various levels of neural plasticity (reviewed in Bartel and Mosabbir, 2021). Music is of course made up of a vast array of sound frequencies and has been shown to have measurable effects on oscillatory activity (Thaut et al., 2005; Fujioka et al., 2015; Kučikienė and Praninskienė, 2018; Yurgil et al., 2020; Lerousseau et al., 2021; Yang et al., 2022; Nandi et al., 2023). Interestingly then, in subjects listening to preferred music, EEG recordings revealed reduced low frequency oscillations associated with lower ratings of pain (Lu et al., 2019), and Gu et al. (2020) reported that, compared to happy or neutral music, sad music was the most effective in alleviating pain, associated with altered brain oscillations in beta and gamma bands. Perhaps linking these studies, the autobiographical recall of sad music results in activation of gamma but also alpha bands (Gupta et al., 2023).

#### Meditation and mindfulness

Evidence presented so far demonstrates the capacity of music to reduce the perception of pain via a number of neuromodulators that are directly involved in modifying ascending nociceptive transmission, at levels ranging from peripheral dorsal root ganglia to cerebral cortex. But music can also regulate other functions such as breathing, relaxation and autonomic nervous system activity, changes typical of altered attentional and conscious states associated with mindfulness and meditation. fMRI and PET studies have compared patterns of brain activation and deactivation associated with mindfulness and different types of meditation (Tang et al., 2015; Fox et al., 2016). Mindfulness meditation was associated with altered activity in the ACC, PFC, insula, striatum (caudate and putamen) and amygdala. In the Fox et al. (2016) review, some differences were seen with different types of practice, but several brain regions showed consistent changes, including the insula, pre/supplementary motor cortices, dorsal ACC and OFC. Overlay of these regions with many of those involved in music processing is evident (Figure 1) and the effects of mindfulness on pain relief appear to be distinct from non-specific effects (Zeidan et al., 2012, 2015; Adler-Neal et al., 2020; Tan and Kuner, 2021).

Reduced pain intensity ratings during mindfulness meditation were associated with increased activity in ACC and anterior insula, while reduced unpleasantness ratings were accompanied by increased activity in OFC and reduced thalamic activity (Zeidan et al., 2011). Others have reported reduced pain-related activity in somatosensory cortex (Nakata et al., 2014). A recent review on the impact of meditation on acute pain also described the involvement of these locations, but included activity changes (some up, some down) in subcortical PAG and RVM (Wipplinger et al., 2023). Importantly, the effectiveness of meditation in reducing acute and chronic pain intensity and unpleasantness varies depending on the experience of the individual (Nakata et al., 2014; Jinich-Diamant et al., 2020). A systematic review of mindfulness-based stress reduction (MBSR) compared to cognitive behavioral therapy (CBT) suggested MBSR could be applied within CBT or in addition to CBT for reducing pain severity, pain interference and psychological distress in chronic pain (Khoo et al., 2019). Other music therapy-based programs involving, for example, cooperative music composition and interaction (Metzner et al., 2022), or a combination of exercise and musical agency (Fritz et al., 2018; Schneider et al., 2022), have also been reported to lessen anxiety and decrease the experience of chronic pain.

As with music-based therapies (Metzner et al., 2022), there is evidence that mindfulness/meditation can enhance parasympathetic activity and HRV (Adler-Neal et al., 2020), but there is an important difference because pain relief is not blocked by opioid antagonists (Zeidan et al., 2016). Indeed, naloxone administration during meditation was shown to enhance pain relief, perhaps by unmasking a non-opioid pathway (May et al., 2018; see also Case et al., 2021). The alternative involvement of GABAergic and dopaminergic systems has been suggested, but there is also now some evidence for increased levels of salivary oxytocin when using mindfulness to reduce anxiety and improve mood (Bellosta-Batalla et al., 2020; Good and Russo, 2022). From the perspective of pain relief and anxiolysis, more research is needed here, especially given earlier studies reporting elevated oxytocin when listening to relaxing (Ooishi et al., 2017) or soothing (Nilsson, 2009) music.

### Some technical caveats and limitations

Measurement of systemic versus CNS levels of classic neurotransmitters, and especially neuropeptides such as β-endorphin and oxytocin, can be affected by technical and interpretative issues. We have already introduced the concept of interactive receptor complexes and modifications, and mention has been made of issues relating to interpretation of receptor occupancy in human PET scans. For many neuromodulators the relative impact of peripheral versus central release is an especially important consideration when elucidating their role in music-induced pain control. Release into the bloodstream does not necessarily correlate with release and activity within the CNS (Veening et al., 2012; Harvey, 2020), and measurements obtained from saliva, plasma or urine are not necessarily reflective of levels in cerebrospinal fluid (CSF), although there is not always agreement as to which peripheral measurements are best (Kagerbauer et al., 2013; Lefevre et al., 2017; Valstad et al., 2017; Martin et al., 2018; Martins et al., 2020).

Much has been made of release kinetics and the half-lives of music-related, antinociceptive molecules such as β-endorphin and oxytocin, and whether half-lives differ in plasma versus CSF. After intravenous (IV) injection of a concentrated bolus of β-endorphin, the mean half-life in plasma was 37 min and after intracerebroventricular injection was 93 min (Foley et al., 1979). Similarly, Aronin et al. (1981) estimated a half-life of 30–50 min, with a small fraction still measurable at 2 h. After IV injection of oxytocin in experimental animals, the half-life was reported to be between 7 to 28 min (Jones and Robinson, 1982; Homeida and Cooke, 1984; Paccamonti et al., 1999), while after intranasal delivery of oxytocin in human subjects, plasma concentrations peaked after 15 min and decreased after 75 min. An increase in CSF concentrations was much slower, with no correlation between CSF or plasma measurements (Striepens et al., 2013). Levels in saliva were also maintained for at least 40 min (Spengler et al., 2017). Of course, while the bioactive half-life of a bolus of exogenously applied peptides provides useful information, the more biologically relevant sustained (perhaps pulsatile) release of endogenous peptides presumably ensures ongoing physiological effects. Finally, there is accumulating evidence that the timing of measurements after exposure to music, the number taken, when during the day the measurements are made, and the mode of extraction and assay technique, can all be critical factors that influence analysis and any interpretation of results (Striepens et al., 2013; Valstad et al., 2017; Boll et al., 2018; Jurek and Neumann, 2018; Martin et al., 2018; Martins et al., 2020; Gan et al., 2023; Tabak et al., 2023).

## Conclusion

Our comprehensive analysis demonstrates that the effect of music on the perception and experience of acute and chronic pain is multifaceted and involves multiple sites and modes of action, both peripherally and centrally. Variability in outcomes is likely related to the type of music that is presented, whether or not the music is part of a music therapy program or selected by the patient or clinician/experimenter, the timing, length and frequency of presentations etc. Furthermore, because both pain and music are multidimensional experiences, the nature of their interaction can be influenced by factors such as distraction, relaxation, expectation, anxiety, and social context. In the CNS there is likely involvement of numerous regions, networks and neurotransmitters/neuromodulators (Figure 1) and we have paid particular attention to dopamine, β-endorphin and oxytocin and the complexity that likely results from interactions of these factors at the receptor and synaptic level. Clinically, the therapeutic use of music has potential to reduce the severe impacts of acute pain in various settings, as a safe adjunct to analgesic and technical medical care. Recognizing the complex multidimensional nature of chronic pain, music has the ability to enhance mood and change the various psychophysiological aspects of pain, not necessarily as a standalone therapy but as part of a multifaceted approach. Unravelling the various mechanisms that underlie the impact of music-based interventions on the human pain experience may aid in the development of better synergistic treatments with other types of antinociceptive therapy, potentially reduce dependency on opiates, and facilitate the development of targeted new agents to treat chronic pain through understanding those physiological mechanisms.

## Author contributions

CA: Writing – review & editing. MB: Writing – review & editing. AH: Writing – review & editing, Conceptualization, Writing – original draft.
